# CORESIDENCE OF OLDER PARENTS AND ADULT CHILDREN BENEFITS OLDER ADULTS’ PSYCHOLOGICAL WELL-BEING: PATH ANALYSIS

**DOI:** 10.1093/geroni/igz038.1181

**Published:** 2019-11-08

**Authors:** Soohyoung r Lee

**Affiliations:** Yeshiva University, New York, United States

## Abstract

Even though the coresidence of older parents and their adult children is no longer a rare phenomenon in current society, a little is known about the benefit of living with adult children from older adults’ perspectives compared to the risk of this living situation. Previous research suggests that older adults’ psychological well-being is low when they live with their adult children, and this become more salient among single parents, such as widowed or divorced. The current paper utilizes the National Health Measurement Study with a sample of age 55 and over, and their SF-36 Mental Health Component score, and psychological well-being self-acceptance score was measured. Path analysis reveals while mental health and psychological well-being scores are lower among single older adults at the time of the survey (e.g., divorced, widowed) than non-single, coresidence of older adults and adult children completely mediates the negative relationship between being single and both mental health psychological well-being. A complete mediation effect of living with an adult child on older adults’ mental health and psychological well-being is consistent with both white and non-white minority older adults. This suggests that living with adult child benefits older adults’ mental health and psychological well-being. The current study seeks to stimulate ideas that might generate the next answer to community-based care in our current aging society.

